# 胸腺肿瘤分子靶向治疗研究现状

**DOI:** 10.3779/j.issn.1009-3419.2014.06.09

**Published:** 2014-06-20

**Affiliations:** 1 200032 上海，复旦大学附属肿瘤医院放疗科，复旦大学上海医学院肿瘤学系 Department of Radiation Oncology, Fudan University Shanghai Cancer Center; Department of Oncology, Shanghai Medical College, Fudan University, Shanghai 200032, China; 2 350014 福州，福建省肿瘤医院 Fujian Provincial Cancer Hospital, Fuzhou 350014, China

**Keywords:** 胸腺上皮肿瘤, 胸腺瘤, 胸腺癌, 靶向治疗, Thymic epithelial tumor, Thymoma, Thymic carcinoma, Target therapy

## Abstract

近年来，随着对分子机制和信号传导通路的深入研究，分子靶向治疗在许多实体瘤中已经取得较大进展，目前已有越来越多的研究关注分子靶向药物在胸腺肿瘤的应用并获得一定经验，分子靶向治疗有可能成为胸腺肿瘤一种新的治疗选择。

## Abstract (en)

In recent years, as the study of molecular mechanism and signal transduction pathways of tumors, molecular target therapy in many solid tumors has made great progress. At present, more and more studies focus on molecular target drugs in thymic epithelial tumors and people have got some experience. Molecular target therapy may be a new therapeutic option for patients of thymic epithelial tumor.

胸腺肿瘤是比较少见的上皮性肿瘤，目前主要治疗方法包括手术、放疗、化疗。手术是主要的治疗手段；不完全切除或者仅做活检的侵袭性胸腺瘤术后放疗地位已经明确；铂类为基础的化疗有一定作用。但对于晚期无法切除、切除后复发或者转移、完全切除后的胸腺肿瘤术后合理的辅助治疗仍没有明确统一的意见。生物靶向治疗以其针对性强、副作用小等优势，逐渐走上肿瘤治疗的舞台，并使部分患者临床获益。与胸腺瘤相关的基因有表皮生长因子受体（epidermal growth factor receptor, EGFR）、Kit、K-ras、Bcl-2、血管内皮生长因子（vascular endothelial growth factor, VEGF）和肿瘤侵袭因子（基质金属蛋白酶和金属蛋白组织抑制剂）等，为靶向治疗提供了分子基础。本文就近年来有关胸腺瘤/胸腺癌分子靶向治疗的研究进行总结和分析。

## 1 c-kit抑制剂

*c-kit*属于生长因子受体类原癌基因，具有酪氨酸激酶活性。在胸腺癌中c-kit过度表达很常见，但在胸腺瘤中很少见^[1]^。Schirosi等^[2]^发现48例胸腺癌患者中，60%过度表达c-kit，但只有6例患者（12.5%）显示*c-kit*突变。

伊马替尼是一种口服靶向抑制c-kit等的多激酶抑制剂。Palmieri等^[3]^用伊马替尼治疗21例晚期胸腺癌患者，结果3例疾病稳定（stable disease, SD），最好的是维持6周稳定的中位缓解期，试验中没有出现4级毒性反应。在治疗的7例B3型胸腺瘤和胸腺癌中，SD 2例，疾病进展（progresive disease, PD）5例，中位生存时间4个月，疾病进展时间中位数为2个月，1/4患者用免疫组化方法检测到c-kit表达，但没有突变^[4]^。另外15例胸腺上皮肿瘤患者（3例胸腺癌），2例免疫组化方法检测到c-kit表达，没有c-kit活性突变，伊马替尼耐受性很好，但没有观察到影像学反应。

## 2 血管生成抑制剂

VEGF的表达水平与肿瘤转移和分期相关^[5]^。人们发现VEGF-A、VEGFR-1和VEGFR-2在胸腺瘤和胸腺癌患者中过度表达^[6]^。然而，血管生成抑制剂对胸腺肿瘤的作用的相关报道比较少。

舒尼替尼是一种口服的多激酶抑制剂，可通过阻断肿瘤生长所需的血液和营养物质供给以及直接攻击肿瘤细胞这两种作用机制来对抗肿瘤。Strobel等^[7]^用舒尼替尼治疗4例难治性胸腺癌，3例部分缓解（partial remission, PR），1例SD，总生存期为4个月-40个月。

索拉菲尼是一种多靶点多激酶抑制剂，抑制血小板衍生生长因子受体（platelet-derived growth factor receptor, PDGFR）、c-Kit和VEGFR等，索拉菲尼的个案报告^[8, 9]^结果显示可能对胸腺瘤（17和11外显子突变）有效。Li等^[10]^报道了1例口服索拉非尼的晚期胸腺癌患者，到报道时已经获得了9个月的稳定，免疫组化显示肿瘤强表达c-kit、p53和VEGF。

SU14813是一种口服的多激酶抑制剂，37例实体肿瘤患者（包括4例胸腺瘤）参与SU14813治疗的Ⅰ期研究^[11]^，12例患者对治疗有阳性反应，包括2例胸腺瘤患者无进展生存期（progression-free survival, PFS）分别持续15.3个月和9个月。

## 3 EGFR抑制剂

EGFR过度表达在胸腺瘤和胸腺癌中很常见，但*EGFR*突变很少见，158例肿瘤患者中只有3例*EGFR*突变^[12]^。荧光原位杂交法检测到的*EGFR*基因扩增较多发生在B3型胸腺瘤和胸腺癌中，与晚期及包膜进展有关。

一项用吉非替尼治疗26例患者（19例胸腺瘤，7例胸腺癌）的Ⅱ期临床试验中，部分缓解（partial response, PR）1例，SD 15例，无完全缓解患者。中位肿瘤进展时间为4个月（1个月-17个月）。不良事件包括呼吸困难、疲劳、贫血、血小板减少、与心肌梗死。

厄洛替尼治疗胸腺瘤有效的报道目前局限在个案报道^[13, 14]^。

一项贝伐珠单抗联合厄洛替尼治疗18例复发的胸腺瘤或胸腺癌患者的Ⅱ期临床试验：11例患者SD，没有出现4级毒性反应，3级毒性包括痤疮样皮疹、呼吸困难、疲劳、心包填塞。

有临床研究^[15, 16]^显示，西妥昔单抗治疗进展期胸腺瘤患者有效，试验中检测到EGFR过度表达，但没有*EGFR*扩增或突变。

目前正在进行的Ⅱ期研究是西妥昔单抗联合CAP（顺铂，阿霉素，环磷酰胺）治疗局部晚期胸腺瘤患者（ClinicalTrials.gov Identifier: NCT01025089），患者接受西妥昔单抗治疗4周，西妥昔单抗+CAP化疗4个周期，而后再进行外科治疗。

## 4 组蛋白去乙酰化酶（histone deacetylase, HDAC）抑制剂

HDAC参与组蛋白的翻译后修饰，从而影响DNA的包装和染色质重塑。HDAC抑制剂通过改变基因表达模式，导致细胞分化、生长停滞，和/或肿瘤细胞凋亡。

个案报道HDAC抑制剂Belinostat^[17]^和MGCD0103^[18]^在治疗转移性胸腺瘤患者中有效。

Ⅱ期临床研究^[19]^显示，41例复发和转移的胸腺瘤或胸腺癌中（25例胸腺瘤，16例胸腺癌），2例PR，25例SD，13例PD，治疗的耐受性良好，恶心、呕吐、疲劳为主要的不良反应。另一项临床试验正在研究Belinostat一线联合CAP方案应用于进展期或者复发的胸腺瘤（NCT01100944）的治疗效果，到目前入组2例患者，治疗的耐受性好，最常见的不良事件是恶心，与化疗本身有关。

## 5 生长抑素（somatostatin, SST）受体

SST受体属于G蛋白偶联受体超家族，在很多肿瘤中表达，包括胸腺上皮肿瘤。早在1990s，人们在胸腺上皮肿瘤组织中发现SST受体，使用放射性标记的奥曲肽能使SST受体显像，用于区分局部晚期和转移疾病。

奥曲肽是一种八肽生长抑素类似物，对选择性的SST亚型受体（SST2）有高亲和力，可能通过阻断胰岛素样生长因子1（insulin-like growth factor 1, IGF-1）或抑制VEGF，发挥在胸腺上皮细胞中的体外抑制作用。东部肿瘤协作组（Eastern Cooperative Oncology Group, ECOG）发起一项奥曲肽（或者联合泼尼松）治疗晚期、无法切除、奥曲肽显像阳性的胸腺肿瘤患者的Ⅱ期研究^[20]^：38例患者（32例胸腺瘤，5例胸腺癌，1例胸腺类癌）入组，2个月评估一次，有反应的继续用奥曲肽治疗，进展的停止治疗。有反应的情况包括2例CR，10例PR，总反应率为32%。另外14例SD，12例PD。38例患者最初都是单独用奥曲肽治疗，只有4例PR。21例患者奥曲肽联合泼尼松，2例CR，6例PR。不良事件包括中性粒细胞减少症、代谢异常、呼吸困难、贫血及白细胞减少症。1年和2年生存率分别是86.6%、75.7%，说明在奥曲肽显像阳性的胸腺瘤患者中单独使用奥曲肽活性有限。泼尼松改善了总生存反应率，但增加了毒性。总之，奥曲肽和泼尼松的整体组合表现出适度的活性，可考虑作为选择性复发患者的治疗方法。而ECOG研究在14例奥曲肽显像阳性胸腺肿瘤患者中使用短效制剂奥曲肽，4例患者有反应，5例SD，2例PD^[21]^。

## 6 IGF-1受体（IGF-1 receptor, IGF-1R）抗体

胸腺癌与胸腺瘤患者中IGF-1R有较高表达水平^[22]^，而IGF-1R表达增加对肿瘤总生存率和疾病进展时间具有较差的预后价值^[23]^。

Figitumumab是一个强有力的、完全的人单克隆IGF-1R抗体，Ⅰ期研究^[24]^发现对转移性胸腺瘤患者有效。

Rajan等^[25]^最近发表了Cixutumumab治疗49例胸腺上皮肿瘤患者（37例胸腺瘤，12例胸腺癌）的Ⅱ期临床试验结果：胸腺瘤组37例患者中5例PR，28例SD，4例PD。胸腺癌组12例患者中无PR，5例SD，7例PD。最常见的3级-4级毒性为血糖升高，血脂升高，体重下降，肿瘤性疼痛和高尿酸血症。

## 7 原肌球蛋白受体激酶（tropomyosin receptor kinase, Trk）和细胞周期蛋白依赖性激酶（cyclin-dependent kinase, CDK）A抑制剂

Trk家族包括TrkA、TrkB和TrkC，它们有内在的酪氨酸激酶活性。TrkA是神经生长因子的特别受体，可能通过MAPK信号通路促进肿瘤生长，胸腺瘤、胸腺癌样本中TrkA拷贝数增加。

CDK可能控制细胞周期G_1_期到S期转录阶段，细胞周期蛋白表达减少，如p21和p27，预示在浸润性胸腺瘤中对新辅助化疗反应较弱^[26]^。基于以上结果，TrkA在胸腺肿瘤中可能是一个重要的治疗靶点。

PHA-848125-AC是一种口服的TrkA、CDK2/Cyclin A抑制剂。一项正在进行的Ⅰ期研究（NCT01011439, NCT01301391）^[27]^发现3例患者中2例（包括B3型和C型）胸腺肿瘤显示为PR。

## 8 类固醇受体辅活化子（steroid receptor coactivator, Src）抑制剂

Src是一种原癌基因编码的非酪氨酸激酶受体。它是Src家族中的一员，参与细胞的增殖和生长、分化、血管生成。Src家族和它的配体被认为在胸腺细胞发展中是重要的成分。

有研究报道了Src抑制剂塞卡替尼（AZD0530）治疗21例晚期胸腺肿瘤患者（14例胸腺瘤和7例胸腺癌）的Ⅱ期研究结果：19例有反应（包括8例SD）。治疗能很好的耐受。3级-4级不良反应包括中性粒细胞减少、贫血、呼吸困难。

达沙替尼是一种新的口服的Bcr-Abl和Src家族激酶等的多激酶抑制剂。有报道^[28]^ABL/Src激酶抑制剂达沙替尼在恶性胸腺瘤中有效。

Hong等^[29]^报道了吉西他滨联合达沙替尼治疗47例晚期实体肿瘤患者Ⅰ期研究结果，3例胸腺瘤患者中2例SD，分别持续9.8个月和15个月。

## 9 总结

综上所述，胸腺上皮肿瘤比较少，临床试验入组有困难，到目前为止，对胸腺瘤/胸腺癌进行靶向治疗的研究虽然局限于个案报道，或者还处于临床Ⅰ期/Ⅱ期试验阶段，但部分药物已显示出治疗效果，且耐受性好、不良反应少，因此，有广阔的研究前景。未来可着眼于组织胸腺瘤/胸腺癌靶向治疗情况的多中心研究，通过这些多中心的前瞻性随机分组对照研究，希望可以解决目前困扰我们的一些分子靶向治疗中的难题。
